# Neutropenic Fever Secondary to Concurrent Clostridioides difficile Infection and Neutropenic Enterocolitis

**DOI:** 10.7759/cureus.86164

**Published:** 2025-06-16

**Authors:** Brandon Wiggins, John M Sullivan, Fady Banno, Kyle Knight, Mark Rigby, Mark Minaudo

**Affiliations:** 1 Gastroenterology, Henry Ford Health System, Grand Blanc, USA; 2 Internal Medicine, Henry Ford Health System, Grand Blanc, USA; 3 Gastroenterology, Corewell Health William Beaumont Hospital, Royal Oak, USA; 4 Gastroenterology and Hepatology, Henry Ford Health System, Grand Blanc, USA

**Keywords:** clostridium difficile, neutropenia, neutropenic enterocolitis, neutropenic fever, typhlitis

## Abstract

Neutropenic enterocolitis (NE), also known as typhlitis, is a life-threatening condition that typically occurs in individuals with severe neutropenia, particularly following recent chemotherapy. It carries a high mortality rate, making rapid identification and treatment essential to prevent serious complications or death. The pathogenesis of NE is not fully understood but is believed to be multifactorial. It involves a sequence of events including cytotoxic drug-induced mucosal injury, microbial invasion of the colonic mucosa, and bowel wall necrosis, all occurring in the context of profound neutropenia, ultimately leading to the clinical manifestation of NE. The resulting colonic wall inflammation makes the bowel highly susceptible to infection by various bacterial and/or fungal pathogens. Common clinical features include neutropenic fever, abdominal pain, diarrhea, and rectal bleeding. Early recognition, initiation of appropriate antibiotic therapy, and supportive care are critical for improving outcomes. In this report, we present the case of a patient with newly diagnosed non-Hodgkin lymphoma who presented with persistent watery diarrhea and was found to have neutropenic fever secondary to concurrent *Clostridioides difficile* infection and NE.

## Introduction

Neutropenic enterocolitis (NE), also known as typhlitis, has an incidence rate of 3.5% among 317 cases of severe neutropenia in individuals older than 16 years and is associated with a mortality rate ranging from 30% to 50% [1,2]. Neutropenia is commonly defined as an absolute neutrophil count (ANC) of less than 1,500/μL and is graded on a scale from mild to severe. When the ANC drops below 500/μL, the neutropenia is considered severe, increasing the risk for NE. The term “typhlitis” is derived from the Greek word “typhi,” meaning “blind,” and refers to inflammation of the “blind”-ending cecum, which typically involves the ileal region and ascending colon as well [3]. NE is characterized by bowel wall edema, thickening, ulceration, and bleeding, and it predominantly affects immunosuppressed patients. Although the pathogenesis is not fully understood, NE is thought to result from intestinal mucosal injury in the context of neutropenia, often induced by cytotoxic chemotherapy or radiation. Depletion of neutrophils is believed to lead to mucosal ulceration, intramural edema, bleeding, necrosis, perforation, and thickening of the gut wall [4]. These features have been identified through gross and histopathological examination of biopsied intestinal mucosa.

The risk of developing NE after chemotherapy may be further increased by preexisting intestinal abnormalities such as diverticulitis, tumor metastasis, or prior abdominal surgery [5]. Therefore, maintaining a high index of suspicion and ensuring early diagnosis are critical. Polymicrobial infections are commonly reported in NE, with the intestinal wall often infiltrated by various bacterial and/or fungal organisms, including gram-negative bacilli, gram-positive cocci, and anaerobes [6]. It is also important to note that patients with neutropenic colitis frequently develop fungal bloodstream infections [5].

In this report, we describe the case of a 68-year-old Caucasian male who developed typhlitis following treatment for newly diagnosed non-Hodgkin lymphoma, along with a discussion on the diagnosis and management of NE.

## Case presentation

A 68-year-old Caucasian male with a past medical history of recently diagnosed diffuse large B-cell lymphoma, type 2 diabetes mellitus, hypothyroidism, and gout presented to the ED after being evaluated by his oncologist earlier that day and was found to be hypotensive, with a reported blood pressure of 70/50 mmHg in the office. Over the preceding two weeks, he had experienced multiple episodes of watery diarrhea, emesis, and generalized weakness following treatment with rituximab, cyclophosphamide, doxorubicin, vincristine, prednisone, and methotrexate for his newly diagnosed non-Hodgkin lymphoma.

In the ED, the patient was initially normotensive but subsequently became hypotensive, requiring multiple fluid boluses. He was febrile, with a maximum temperature of 103°F prior to admission, and was also tachycardic and tachypneic. Laboratory evaluation revealed leukocytopenia with an ANC of zero, thrombocytopenia, lactic acidosis, multiple electrolyte abnormalities, acute kidney injury, and hypoalbuminemia. Empiric antibiotic therapy with vancomycin and piperacillin-tazobactam was initiated.

**Figure 1:**
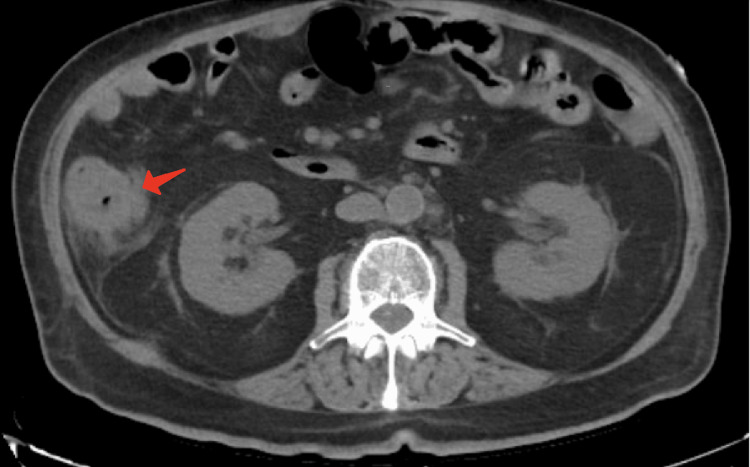
CT scan of the abdomen and pelvis showing inflammation of the cecum and ascending colon, findings consistent with typhlitis

A broad workup for sepsis ruled out pneumonia and urinary tract infection; however, blood cultures were positive for *Klebsiella pneumoniae *and *Proteus mirabilis*. Given these findings and the patient’s clinical status, a CT scan of the abdomen and pelvis with IV and oral contrast (Figure 1) was performed, revealing inflammation of the ascending colon and cecum consistent with typhlitis, presumably secondary to *K. pneumoniae *and *P. mirabilis*. Stool studies were positive for *Clostridioides difficile*, with detection of both glutamate dehydrogenase antigen and pathogenic loci.

Following consultation with the infectious disease team, concern for extended-spectrum beta-lactamase (ESBL) bacteremia was raised. Consequently, vancomycin and piperacillin-tazobactam were discontinued, and antimicrobial therapy was adjusted to include meropenem, anidulafungin, metronidazole, and oral vancomycin. The oncology team also evaluated the patient and initiated treatment with filgrastim and platelet transfusions.

The patient’s blood culture sensitivities eventually returned, ruling out ESBL-producing organisms; consequently, antibiotics were de-escalated to ceftriaxone, metronidazole, and oral vancomycin. The patient’s clinical status improved following this treatment regimen, and repeat blood cultures and *C. difficile *testing were negative prior to discharge.

## Discussion

NE, or typhlitis, should be strongly considered in the differential diagnosis of a cancer patient presenting with neutropenic fever following recent chemotherapy, as it is a life-threatening condition requiring prompt intervention. Also referred to as “necrotizing enterocolitis” [7], the pathogenesis of NE remains incompletely understood, though it is believed to be multifactorial. Contributing factors include cytotoxic drug-induced mucosal injury resulting in mucositis, necrosis of the bowel wall, and profound neutropenia. Common pathogens associated with NE include *Pseudomonas aeruginosa*, *Escherichia coli*, *Viridans streptococci*, *Clostridium *spp., *Candida *spp., *Bacteroides *spp., and *Klebsiella *spp. [5,8], and antibiotic therapy should be guided by the need to cover these organisms.

Risk factors for NE include prolonged neutropenia, chemotherapy within two weeks of symptom onset, hematopoietic cell transplantation, and the presence of mucositis. A 2007 report documented the incidence of typhlitis as 3.5% among 317 episodes of severe neutropenia and fever in individuals older than 16 years [1]. Clinicians should maintain a high level of suspicion for NE in any neutropenic patient presenting with fever and gastrointestinal symptoms such as diarrhea, cramping, bleeding, abdominal distension, and reduced oral intake.

Neutropenic fever is a key diagnostic consideration in suspected NE, though definitions may vary slightly among clinical guidelines [9]. According to the European Society for Medical Oncology, febrile neutropenia (FN) is defined as a single oral temperature >38.5°C or two consecutive readings >38.0°C over two hours, combined with an ANC <0.5 × 10⁹/L, or an ANC expected to fall below this threshold [10]. Although FN can result from bacterial, fungal, or viral infections, bacterial pathogens are the most common cause [11]. Currently, gram-positive organisms are responsible for approximately 60% of FN cases, while gram-negative organisms account for about 40% [12,13].

When evaluating a patient with suspected neutropenic fever, a thorough history and physical examination are essential, along with laboratory testing that includes a complete blood count with differential, electrolytes, liver enzymes, serum creatinine, bilirubin, serum lactate, and blood urea nitrogen. Infectious workup should include blood cultures and urinalysis. In patients with diarrhea, *C. difficile* toxin assay and stool cultures for ova and parasites should be performed. If respiratory symptoms are present, sputum samples should be evaluated for bacterial, fungal, and viral pathogens [14]. In high-risk patients, such as those with hematologic malignancies or undergoing hematopoietic stem cell transplantation, additional tests, including Aspergillus galactomannan antigen and beta-D-glucan assay, are recommended [15]. For suspected meningitis, and in the absence of contraindications, cerebrospinal fluid should be analyzed for glucose, protein, bacterial culture, and PCR for viruses such as herpes simplex virus, cytomegalovirus, varicella-zoster virus, and human herpesvirus 6. Chest radiographs should be obtained in low-risk patients to evaluate for pneumonia, while high-risk patients should undergo a non-contrast CT of the chest.

Treatment of NE generally includes bowel rest, nasogastric suction, IV fluids, blood product support, and broad-spectrum antibiotics. Crucially, empiric antibiotic therapy should be initiated immediately, ideally within 60 minutes of hospital presentation and after blood cultures have been obtained. First-line antibiotic therapy should include an antipseudomonal beta-lactam agent. Vancomycin may be added if gram-positive coverage is warranted, such as in the presence of a central venous catheter. Antibiotic regimens should target* P. aeruginosa*, *E. coli*, enteric gram-negative bacilli, and anaerobes. Recommended regimens include piperacillin-tazobactam or cefepime plus metronidazole. Carbapenems may be considered in patients with beta-lactam allergies or colonization with resistant organisms. In patients with persistent fever lasting more than 72 hours despite antibiotic therapy, empiric antifungal coverage should be added. Treatment should continue until neutrophil recovery and clinical resolution of NE symptoms.

As noted, NE carries a reported mortality rate of 30% to 50% [2] and demands rapid diagnosis and intervention. Poor prognostic outcomes are typically linked to complications such as transmural necrosis, bowel perforation, and sepsis [16]. Given the potential for rapid deterioration, involvement of critical care and surgical teams may be necessary. Close collaboration among critical care, surgical, and nursing staff is essential to optimize supportive care and promote recovery.

## Conclusions

NE, or typhlitis, is a life-threatening condition that can develop in individuals with neutropenia who have recently undergone chemotherapy. It is most commonly associated with cases of severe neutropenia, with a reported incidence of 3.5%. The pathogenesis is multifactorial, involving cytotoxic drug-induced mucosal injury, subsequent invasion of the damaged mucosa by gut microorganisms, bowel wall necrosis, and profound neutropenia. As a result, the intestinal wall becomes highly susceptible to infiltration by various bacterial and/or fungal organisms, and polymicrobial infections are frequently observed. A thorough clinical history is essential to guide the clinician in ruling in or ruling out NE. Commonly reported symptoms include neutropenic fever, diarrhea, abdominal cramping, gastrointestinal bleeding, distension, and reduced oral intake. Clinicians must maintain a high index of suspicion for NE in any neutropenic patient presenting with such symptoms. Prompt abdominal and pelvic CT imaging with both oral and IV contrast is strongly recommended to aid in diagnosis. Early recognition, timely initiation of broad-spectrum antibiotics, and supportive care are critical to improving patient outcomes. Clinicians should routinely consider NE in the differential diagnosis of neutropenic fever and tailor empiric antibiotic therapy to include coverage for organisms commonly implicated in NE, given the high mortality rate, up to 50%, if the condition is not addressed promptly. A multidisciplinary approach should be implemented early in the clinical course to provide comprehensive care and ensure lifesaving interventions are available should the patient’s condition rapidly deteriorate, as is often the case.
